# Reducing the life cycle environmental impact of electric vehicles through emissions-responsive charging

**DOI:** 10.1016/j.isci.2021.103499

**Published:** 2021-11-22

**Authors:** Yuzhou Tang, Tim T. Cockerill, Andrew J. Pimm, Xueliang Yuan

**Affiliations:** 1 Research Center for Sustainable Development, School of Energy and Power Engineering, Shandong University, 17923 Jingshi Road, Jinan 250061, China; 2 School of Mechanical Engineering, University of Leeds, Leeds, LS29JT, UK; 3 School of Chemical and Process Engineering, University of Leeds, Leeds, LS29JT, UK

**Keywords:** EV charging, Charging behavior cluster, Emissions factors, Demand side response, Life cycle assessment

## Abstract

Electric vehicles (EVs) are currently being promoted to reduce transport emissions. We present a life cycle assessment of EV charging behaviors based on marginal emissions factors. For Great Britain, we find that electricity consumption accounts for the highest proportion of life cycle carbon emissions from EVs. We highlight the potential life cycle carbon emissions reduction brought by charging during periods when the grid mix produces relatively low emissions. While our study focuses on Great Britain, we have applied our methodology to several European countries with contrasting electricity generation mixes. Our analysis demonstrates that countries with a high proportion of fossil energy will have reduced benefits from deploying EVs, but are likely to achieve increased benefits from smart charging approaches. We conclude that using marginal emissions factors is essential to understanding the greenhouse gas impacts of EV deployment, and that smart charging tied to instantaneous grid emissions factors can bring benefits.

## Highlights

• Propose LCA methodology for EV charging behaviors
• Marginal emissions factors are used to improve LCA accuracy
• Smart charging could reduce emissions by up to 6% for certain charging event
• Plug-in duration is found to have a crucial impact on smart charging performance

## Graphical abstract

**Figure:**
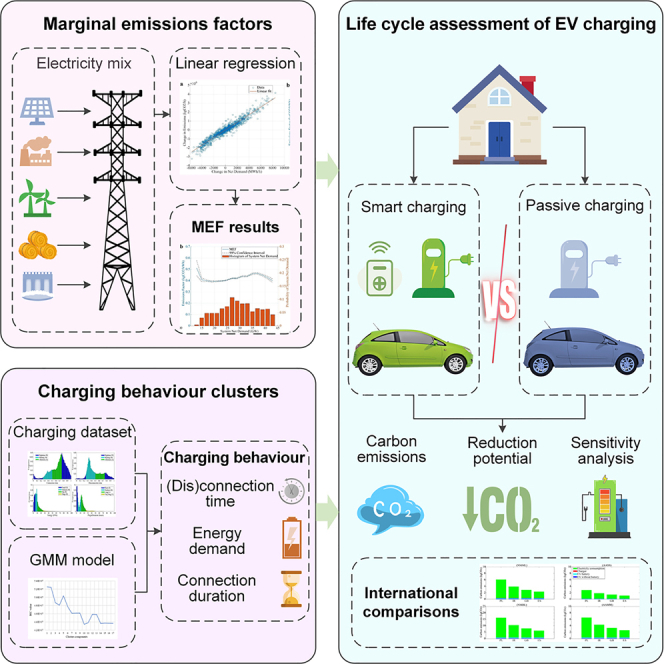

## Teaser

EV charging, Charging behaviour cluster, Emissions factors, Demand side response, Life cycle assessment

## Introduction

Electric vehicles (EVs) are set to gradually replace internal combustion engine vehicles (ICEVs) as the main technology for personal transportation (OLEV, Office for Low Emission Vehicles, 2017). This transformation is intended to reduce greenhouse gas (GHG) emissions significantly over vehicle lifetimes, supporting net-zero targets (BEIS, The Department for Business, Energy and Industrial Strategy, 2019b). Meanwhile, the electrification of passenger vehicles is considered a promising strategy to reduce the environmental impact of transportation (Hill, et al., 2019), which mainly includes battery electric vehicles (BEVs), plug-in hybrid electric vehicles (PHEVs), and fuel cell electric vehicles (FCEVs) (Boriboonsomsin, 2021). In this study, we investigate the extent to which EV's reduce emissions, and explore how smart vehicle charging processes can be managed to maximize the potential benefits. The assessed EVs include BEVs and PHEVs.

Several previous studies have suggested that introducing EVs may not necessarily lead to carbon emissions reductions in every case (Del Pero et al., 2018; McLaren et al., 2016; Casals et al., 2016; Buchal et al., 2019), and in some circumstances EV emissions could exceed those of ICEVs. The lifecycle analysis (LCA) approaches used in these studies indicate that the origin of the electricity used for charging is key, with carbon intensity being a critical factor (Petrauskienė et al., 2020; Raugei and Winfield, 2019; Rosenfeld et al., 2019; Wu et al., 2019; Burchart-Korol et al., 2020). In many studies, however, the carbon intensity of electricity is evaluated in a relatively simple manner that does not take full account of its increasingly important variation with time and location (Rees et al., 2014; Sun et al., 2019).

Marginal emissions factors (MEFs) provide a convenient approach for characterizing the temporal variation in the carbon intensity of the electricity used to charge EVs. While MEFs are well understood in power system analysis, their application to the LCA of EVs has received scant attention in the literature (Hawkes, 2014; McLaren et al., 2016; Pimm et al., 2019; Jochem et al., 2015). This paper further develops an MEF-based technique formulated by some of the authors to evaluate how smart charging approaches can be used to minimize the emissions associated with EV charging. As MEFs vary with time, charging behavior (i.e., when users choose to charge their vehicles) plays a crucial role in influencing emissions (see e.g., Morrissey et al., 2016). We supplement our approach therefore with a specific cluster analysis of EV domestic charging behavior, accounting for charging time, plug-in duration, and energy demand. This cluster analysis builds on concepts from charging behavior modeling efforts (Sun et al., 2020; Aghabozorgi et al., 2015; Khaki et al., 2019; Akman et al., 2019; Xydas et al., 2016; Straka and Buzna, 2019) and the identification of charging characteristics (Chaouch, 2014; Helmus et al., 2020; Wang et al., 2019).

At the conclusion of our analysis, a comparative environmental impact assessment of EV charging is presented. The carbon emissions from different charging behaviours are assessed using hourly electricity grid emissions factors, and we determine the potential to further reduce emissions by load shifting during the period the vehicle is plugged in. Our work provides insights into the carbon emissions associated with EVs in the UK, considering upstream emissions from electricity generation, and analyses the decarbonization potential of smart EV charging. As electricity generation mixes vary considerably between different countries, we also present comparative research for Poland, Ireland, and Spain, demonstrating how the methodology can be applied in different national contexts.

## Results and discussion

### Baseline life cycle carbon emission analysis using average emissions factors

**Figure 1:**
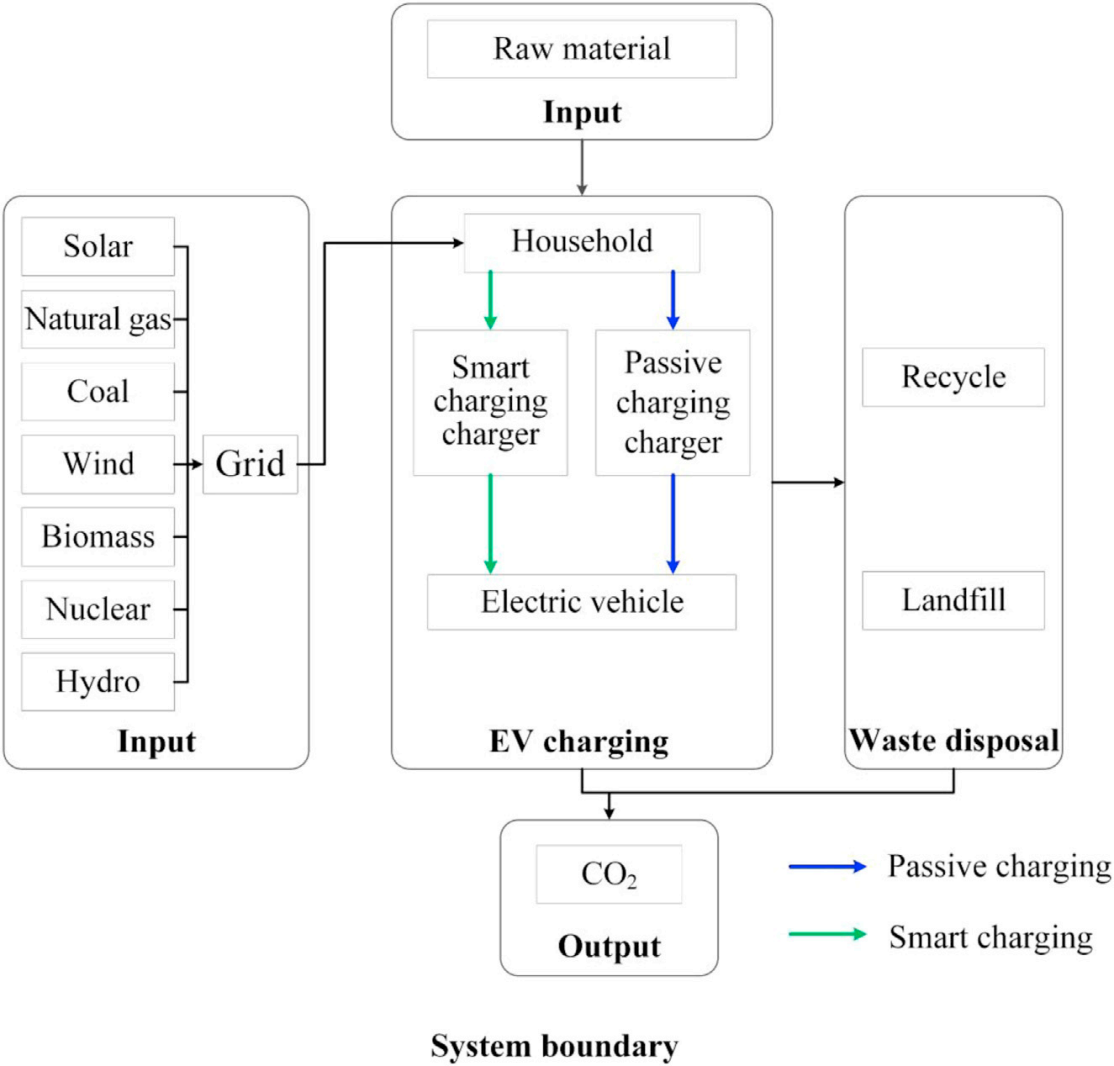
System boundary of the research

To provide background, and give a baseline against which to demonstrate the importance of using MEFs to analyse charging emissions, we present a simple analysis of the life cycle carbon emissions associated with EV charging in British households for 2019. For this analysis, we assume that an EV starts charging immediately when it is plugged in, and continues charging until the battery is fully charged, a process we will refer to as “passive charging”. The functional unit of the system is taken as a single domestic EV charging event, and all analyses use the same functional unit for comparison. The system boundary is defined as “cradle to grave,” as shown in Figure 1.

**Figure 2:**
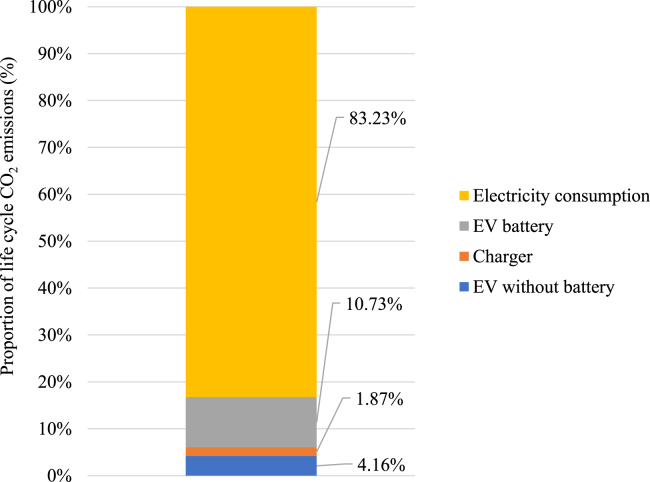
Life cycle carbon dioxide emissions for EV charging

Figure 2 provides the calculated life cycle carbon emissions for the EV, in which the average carbon emissions factors (AEFs) for the GB electricity grid in 2019 as given in the greenhouse gas reporting (BEIS, The Department for Business, Energy & Industrial Strategy, 2019a) are used to calculate the environmental impact of electricity consumption. The results show that the life cycle carbon emissions are primarily related to the energy delivered. Electricity consumption accounts for approximately 83% of the total CO_2_ emissions, followed by manufacturing of the battery (11%), the EV without battery (4%), and the charger (2%).

Clearly, electricity consumption is the key source of carbon dioxide emissions associated with this EV. However, as with most previous research, the analysis in this section has not taken variations in grid emissions factors into account. In practice, carbon emissions can vary considerably over time depending upon the share of generation from renewables, and the time at which charging takes place is the key determinant of life cycle carbon emissions. In this paper, we explore the effect of taking this variation into account.

### Clustering of charging behaviors

To investigate the greenhouse gas emissions associated with EV charging, and the potential to reduce these emissions by load shifting in response to grid emissions factors, we make use of the domestic EV charging dataset provided by the UK government's office for low emission vehicles (OLEV), focusing on the characteristics of charging time, charging duration, and energy demand (DfT, The Department for Transport, 2018). This dataset comprises over 3 million charging sessions, so clustering is used to focus the analysis on a small number of representative charging sessions. Our clustering approach draws on previous work aimed at analyzing large charging behavior datasets (Helmus et al., 2020).

**Figure 3:**
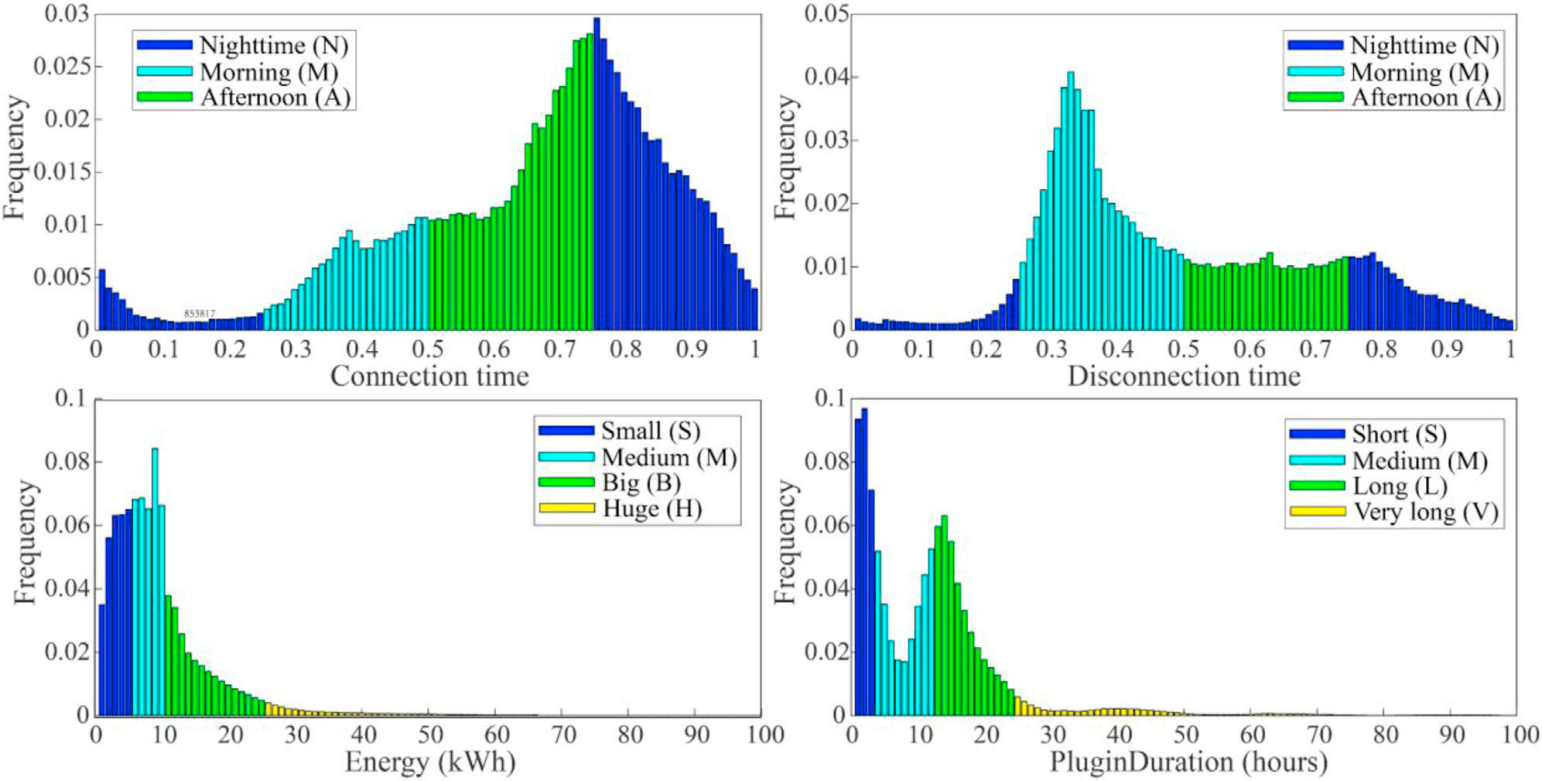
Features of charging sessions

We consider a charging session to be a decision of an EV user to charge a specific volume of energy from a particular connection time, for a specific duration, and then disconnect at a specific time. Histograms of the four features (connection time, disconnection time, energy consumed, and connection duration) of sessions in the OLEV dataset are shown in Figure3.

For each feature shown in the figure, colours are used to indicate the groups used in the clustering process. Connection and disconnection times were normalized from 0 to 1, with 0 and 1 indicating the start and end of the 24-h day (00:00 and 23:59, respectively). Connection and disconnection times were divided into morning (06:00–12:00), afternoon (12:00–18:00), and night time (18:00–06:00) periods for clustering purposes, represented by the letters M, A, and N, respectively. The connection and disconnection times are characterized by evening and morning peaks, respectively. The energy consumption features were divided into four groups: small (0–5 kWh), medium (5–10 kWh), big (10–25 kWh), and huge (>25 kWh), represented by the letters S, M, B, and H, respectively. The consumption data are mainly concentrated in the small and medium groups, with the number of charging sessions at higher levels of consumption tending to zero. The plug-in duration was separated into four groups: short (0–3 h), medium (3–12 h), long (12–24 h), and very long (>24 h), represented by the letters S, M, L, and V, respectively. The distribution of plug-in duration contains two distinct peaks, one in each of the short and long connection duration groups.

**Figure 4:**
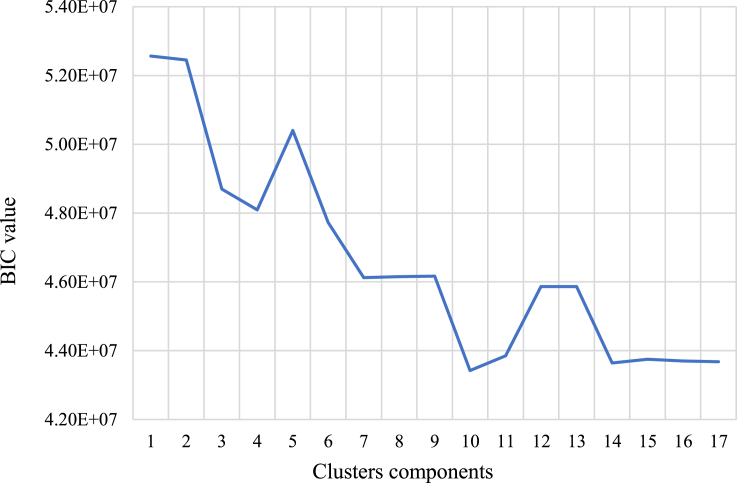
Result of BIC analysis for charging sessions in 2017

The Gaussian mixture model (GMM) approach was used to develop clusters of charging events based on these four features. Using the Bayesian information criterion (BIC), it was found that a cluster size of 10 provides the best model performance, as shown in Figure 4. The BIC value represents the overfitting of the model, and the lower value indicates better fitted model. The details of the four features for each cluster, labelled using the first letters of the feature names, are presented in Table 1. A large majority of sessions (over 90%) are in the clusters NMML, AAMM, AASS, and NMBL. Two of these (NMML and NMBL, containing over 50% of sessions) are characterized by overnight charging, with connection and disconnection times being around the end and start of the work day. The other two (AAMM and AASS) are characterized by afternoon charging. These four clusters are the focus for the rest of this study.

**Table 1:** Overview session types, abbreviations, and distribution properties

Abbreviation	Connection time	Disconnection time	Energy	Connection duration	Sessions
AABV	Afternoon,	Afternoon,	Big,	Very long,	4,212
	15:10,	12:22,	18.2 kWh	740.0 h	0.13%
	δ5.5 h	δ5.4 h	δ17.1 kWh	δ721.6 h	
AMBV	Afternoon,	Morning,	Big,	Very Long,	18,652
	15:20,	11:48,	22.1 kWh	125.7 h	0.59%
	δ5.3 h	δ4.8 h	δ17.6 kWh	δ51.0 h	
MABL	Morning,	Afternoon,	Big,	Long,	51,626
	09:43,	14:24,	24.2 kWh	15.3 h	1.63%
	δ6.2 h	δ5.2 h	δ6.1 kWh	δ9.1 h	
NAML	Nighttime,	Afternoon,	Medium,	Long,	52,562
	03:30,	13:40,	7.5 kWh	20.6 h	1.65%
	δ3.4 h	δ5.7 h	δ4.0 kWh	δ9.4 h	
AMHV	Afternoon,	Morning,	Huge,	Very long,	64,683
	16:09,	10:22,	30.7 kWh	41.0 h	2.04%
	δ5.5 h	δ4.2 h	δ19.8 kWh	δ24.5hrs	
AMMV	Afternoon,	Morning,	Medium,	Very long,	81,508
	16:52,	10:37,	8.6 kWh	41.5 h	2.57%
	δ4.0 h	δ3.8 h	δ4.8 kWh	δ5.4 h	
NMBL	Nighttime,	Morning,	Big,	Long,	402,293
	19:21,	08:13,	19.2 kWh	12.9 h	12.66%
	δ2.4 h	δ2.1 h	δ6.9 kWh	δ3.0 h	
AASS	Afternoon,	Afternoon,	Small,	Short,	568,331
	13:54,	15:00,	3.4 kWh	1.1 h	17.89%
	δ4.1 h	δ4.1 h	δ1.9 kWh	δ0.6 h	
AAMM	Afternoon,	Afternoon,	Medium,	Medium,	690,071
	12:28,	16:25,	8.0 kWh	3.9 h	21.72%
	δ4.5 h	δ4.2 h	δ4.7 kWh	δ2.0 h	
NMML	Nighttime,	Morning,	Medium,	Long,	1,242,865
	18:11	09:02	7.22 kWh	14.85 h	39.12%
	δ3.20 h	δ3.05 h	δ3.04 kWh	δ4.47 h	

For connection and disconnection times.

Morning time (M): 06:00–12:00; Afternoon time (A): 12:00–18:00; Nighttime (N): 18:00–06:00.

For energy.

Small (S): 0–5 kWh; Medium (M): 5–10 kWh; Big (B): 10–25 kWh; Huge (H): >25 kWh.

For connection duration.

Short (S): 0–3 h; Medium (M): 3–12 h; Long (L): 12–24 h; Very Long (VL):>24 h.

### Life cycle carbon emissions with marginal emissions factors

**Figure 5:**
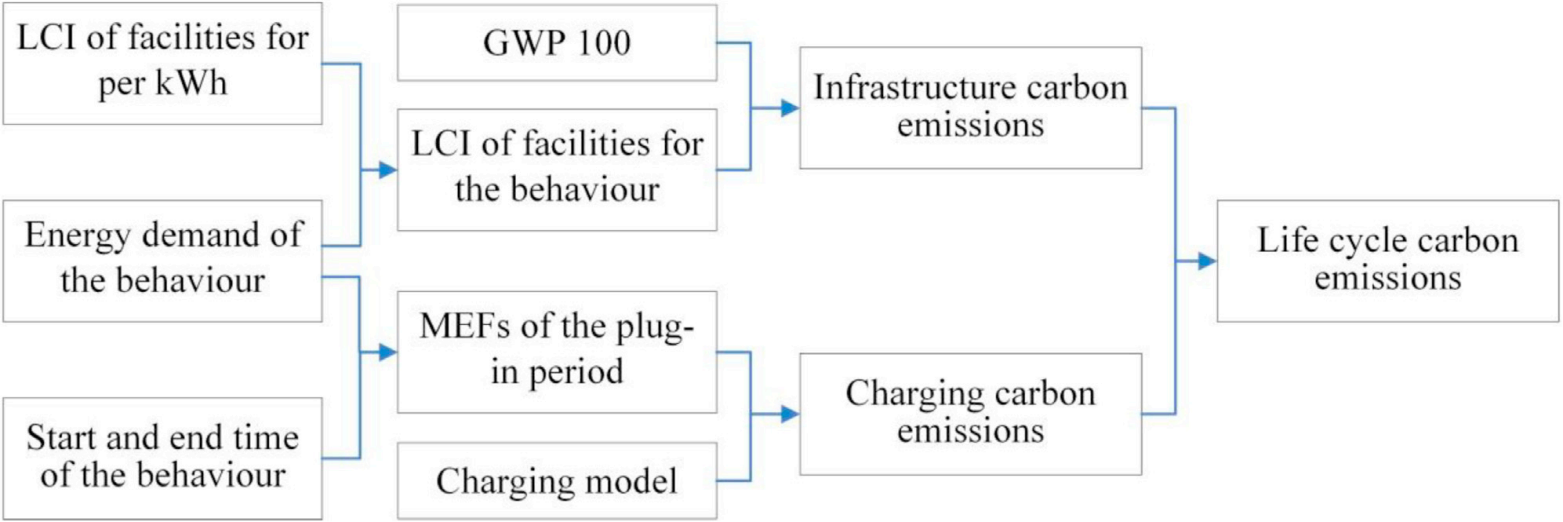
Flow chart of the life cycle carbon emissions calculation

For our analysis, the life cycle carbon emissions associated with a charging event are divided into two parts: facilities (battery, charger, and EV without battery) and electricity consumption, as shown in Figure 5. We focus on the four major clusters (NMML, AAMM, AASS, and NMBL) only. The connection and disconnection times for each cluster are used to calculate the carbon emissions from the grid with the MEF analysis. The carbon emissions of the four main behavior clusters under the functional unit (a single domestic EV charging event) are calculated for each day of the year to reveal the effect of variations in grid emissions factors over the year.

**Figure 6:**
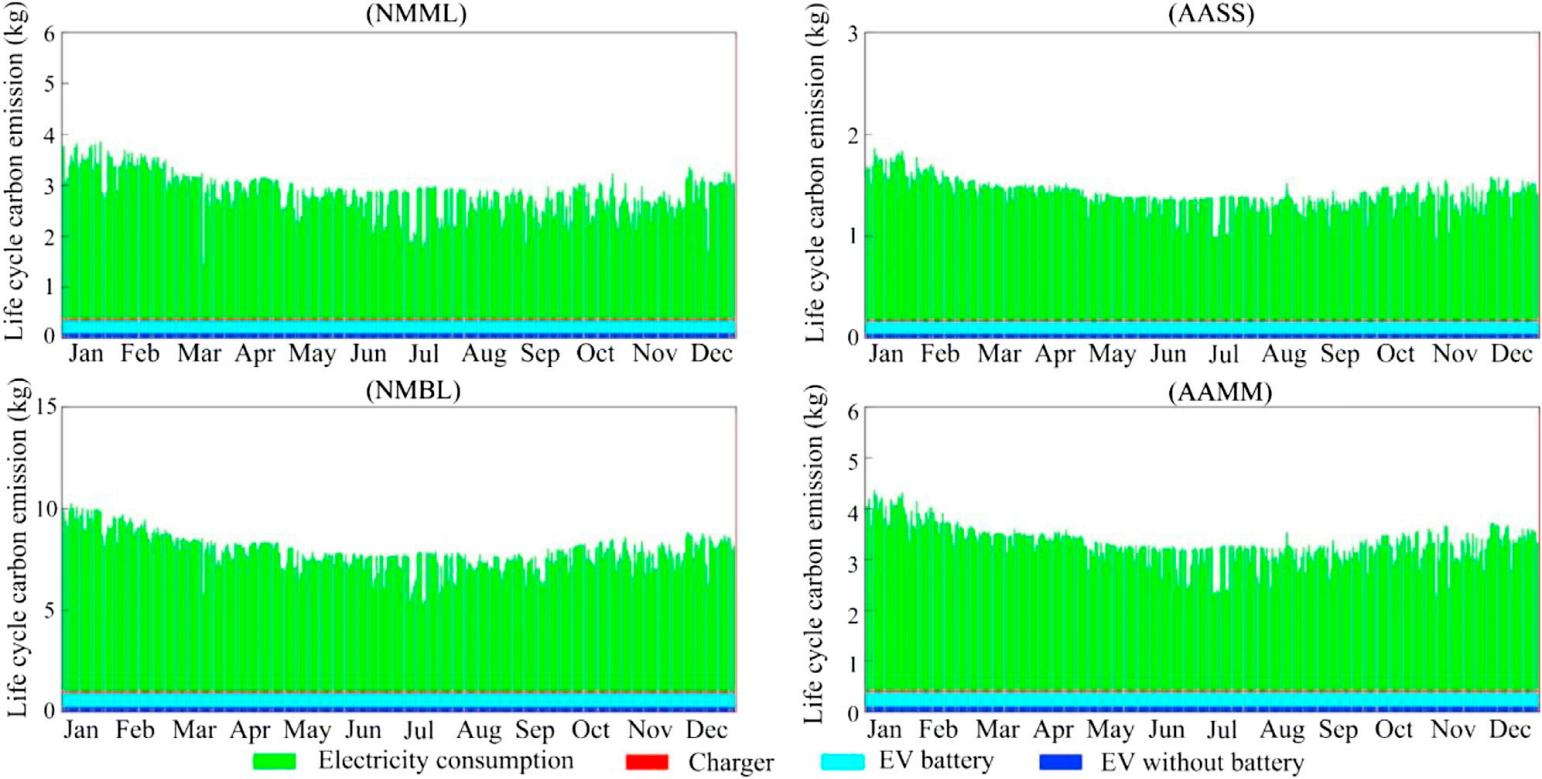
Life cycle carbon emissions for each charging behavior in 2019

Figure 6 provides a complete display of the life cycle carbon emissions associated with every charging event for the four main behavior clusters from 1st January 2019 to 31st December 2019. A summary of the life cycle carbon emissions from one charging event for each charging behavior is shown in Table 2. The life cycle carbon emissions calculated using MEFs are 25% higher than those found using the AEFs for the GB grid in 2019, which is similar to the difference found by Hawkes (Hawkes, 2010) in an analysis of the national energy system. In addition, many studies have shown that AEFs would seriously erroneously calculate the emissions related to the intervention (Hawkes, 2010; Bettle et al., 2006; Thind et al., 2017). Therefore, MEFs are more reliable than AEFs in evaluating the carbon emissions impact in this study.

**Table 2:** Life cycle carbon emissions of one charging event for each behavior through 2019 (in kgCO_2_)

	NMML	AASS	NMBL	AAMM
Minimum	1.8	1.0	6.3	2.7
Maximum	4.3	1.9	11.3	4.8
Mean	2.9	1.4	7.9	3.3
Based on AEFs	2.3	1.1	6.2	2.6

Upstream emissions from electricity generation account for the large difference in emissions for one charging event across different times of the year. The results show that variations in upstream emissions could lead to more than a 50% life cycle carbon emissions difference (for example, considering NMMLbehaviour, the emissions of the charging event on January 21 emits double that of the charging event on March 18). The reason behind this should be the difference in electricity generation structure during the same period of these two days. Carbon emissions in wintertime (December, January, and February) are higher than those in summertime (June, July, and August), and the difference in average emissions between the two periods is approximately 20%.

### Potential carbon emissions reduction from EV smart charging

Electricity consumption is responsible for the majority of the carbon emissions from EV charging in our functional unit. We now investigate the potential to reduce these emissions by changing the charging time point from times of high MEF to times of low MEF, known here as smart charging. The emissions reduction associated with this approach are calculated through comparison with passive charging. One hour is the minimum length of a charging period according to the data resolution. As a result, the AASS cluster does not have the possibility to reduce emissions by load shifting. Therefore, the clusters of NMML, NMBL, and AAMM are analysed with both passive and smart charging approaches, but only passive charging is analysed for AASS.

**Figure 7:**
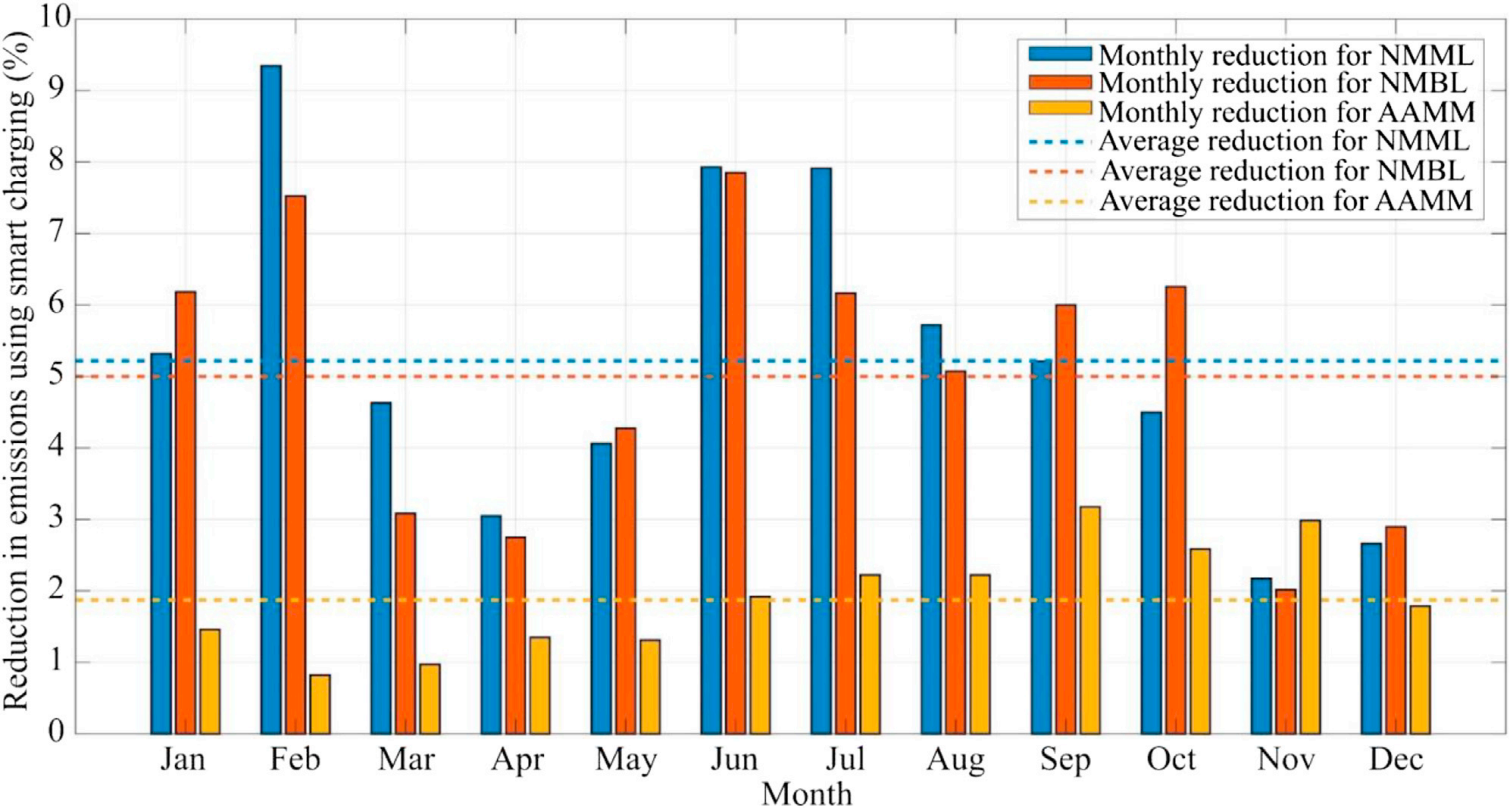
Percentage reduction of the NMML, NMBL, and AAMM behaviors from using smart charging of EVs instead of passive charging

Figure 7 shows the monthly average reductions in life cycle carbon emissions of the NMML, NMBL, and AAMM behaviors. The decarbonization potential is greatest for the NMML and NMBL behaviors because both are characterized by overnight charging and hence long idle times. These provide increased flexibility, demonstrating the highest average life cycle carbon emissions reduction (around 5% in both cases). AAMM has a medium plug-in duration and could only achieve a reduction in life cycle carbon emissions of approximately 2%. The charging events in the AASS behavior are only one hour long; therefore it is not possible to determine the benefits of smart charging using hourly resolution MEF data. The results illustrate that the benefits of smart charging are highly associated with the plug-in duration.

Emissions reduction from smart charging varies considerably with month. For example, compared with passive charging, smart charging for NMML in February can induce a more than 9% life cycle emissions reduction, while that in November could give only a 2% reduction. Because of the behavior characteristics of the connection and disconnection time, NMML and NMBL have the similar reduction trend in months, but AAMM is different. The decarbonization benefits mainly rely on the low MEFs time during the plug-in duration of charging behaviors.

Compared with passive charging, smart charging achieves around a 6% reduction in carbon emissions when used in the most common charging behaviors, characterized by overnight charging. Plug-in duration was found to have a crucial impact on smart charging performance, with longer connections typically providing greater opportunity for load shifting and wider variations in grid emissions factors.

The benefits of smart charging are related to the charge rate as well as charging behaviors. We assume that the battery controller of the vehicle does not limit charge rates, so that the impact of different domestic charger capacities (3–22 kW) can be analysed.

**Figure 8:**
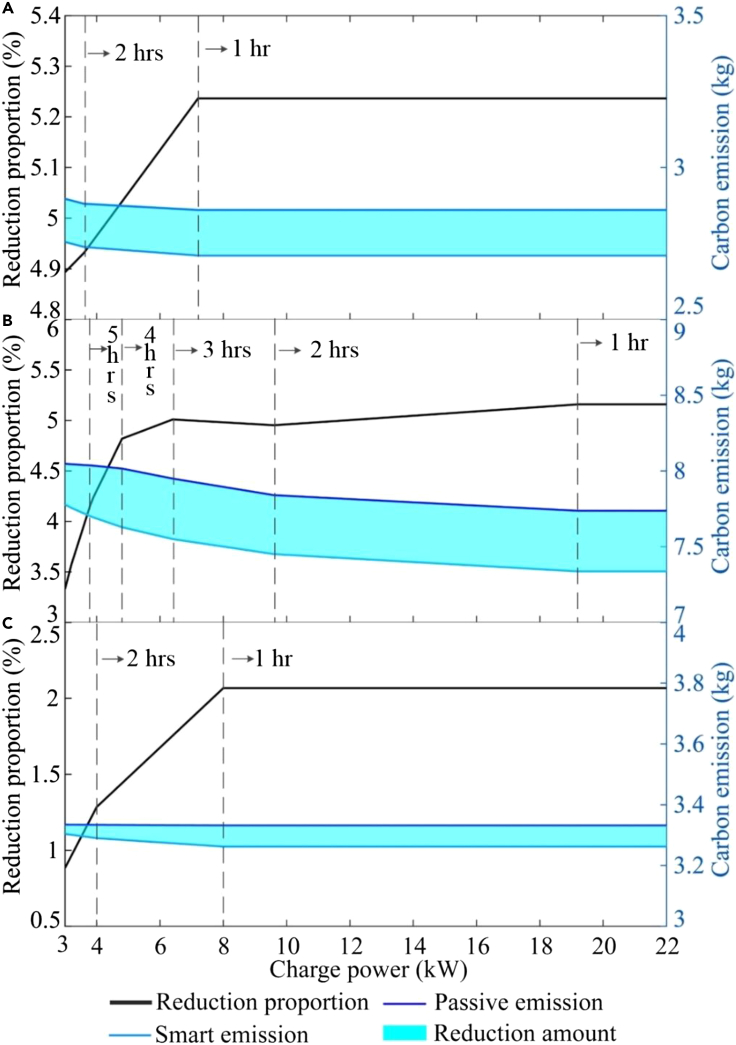
Sensitivity analysis of charge rate (A) Sensitivity for NMML. (B) Sensitivity for NMBL. (C) Sensitivity for AAMM. The time that it would take to fully charge the battery from 0% state of charge with the different charger powers is indicated using vertical dashed lines.

The change in charging flexibility is apparent (Figure 8). Increasing charger capacity allows the required energy to be delivered in a shorter period, increasing the idle time and allowing more charging to occur in the lowest MEF periods when smart charging is used. Thus, the carbon emissions reduction curve for different charging behaviors has some intersection points. This phenomenon could be due to the hourly MEF data used in the present study, with the intersection points reflecting the change in charging hours. The proportion of emissions reduction for NMML and AAMM steadily increases until the charging time is less than one hour. Considering NMBL, the reduction proportion increases significantly with charge rate when the charge rate is below 6.4 kW; above this, it rises more gradually with charge rate to 5%.

Overall, sensitivity analysis revealed that increased charge rate improves the benefits of smart charging by introducing flexibility to the charge times, and this is particularly apparent when charge rate is increased from an otherwise low level. Charger capacities of 7 kW could generally achieve good emissions reductions through smart charging at households.

### International comparisons

To explore how the results vary in other countries, the same approaches are now applied to the national electricity systems of Poland (PL), Ireland (IR), and Spain (ES), to compare the greenhouse gas emissions from charging electric vehicles with the emissions from internal combustion engine vehicles and assessed the potential to reduce electric vehicle charging emissions through smart charging in these countries with each other and Great Britain. Table 3 shows the structure of the electricity system in the different countries of interest. We selected these countries to explore the effect of different dependencies on fossil fuel-derived electricity.

**Table 3:** Proportion of total energy supply for different generation types and the average MEFs of four countries in 2019

	Great Britain (GB)	Spain (ES)	Poland (PL)	Ireland (IE)
CCGT	41.1%	29.0%	6.8%	35.3%
Oil	0	0.8%	1.0%	8.9%
Coal	2.1%	4.2%	73.4%	11.2%
Nuclear	19.0%	21.1%	0	0
Wind	16.5%	19.7%	9.3%	39.3%
Pumped	0.6%	1.2%	0.6%	2.5%
Hydro	1.3%	10.1%	1.0%	3.4%
Other	0.3%	0.5%	0	0
Biomass	6.2%	1.1%	1.3%	0
Solar	4.0%	5.4%	0	0
Transmission	9.0%	5.9%	6.6%	−0.1%
Average MEFs (g/kWh)	361	276	761	492

Poland has the highest proportion of fossil energy (more than 80%), followed by Ireland (53%), Great Britain (43%), and Spain (34%). Based on the hourly generation and demand data, the MEFs for Poland, Ireland, and Spain were calculated using the same approach as Great Britain. The MEFs are predominantly positively correlated to the proportion of fossil energy.

**Figure 9:**
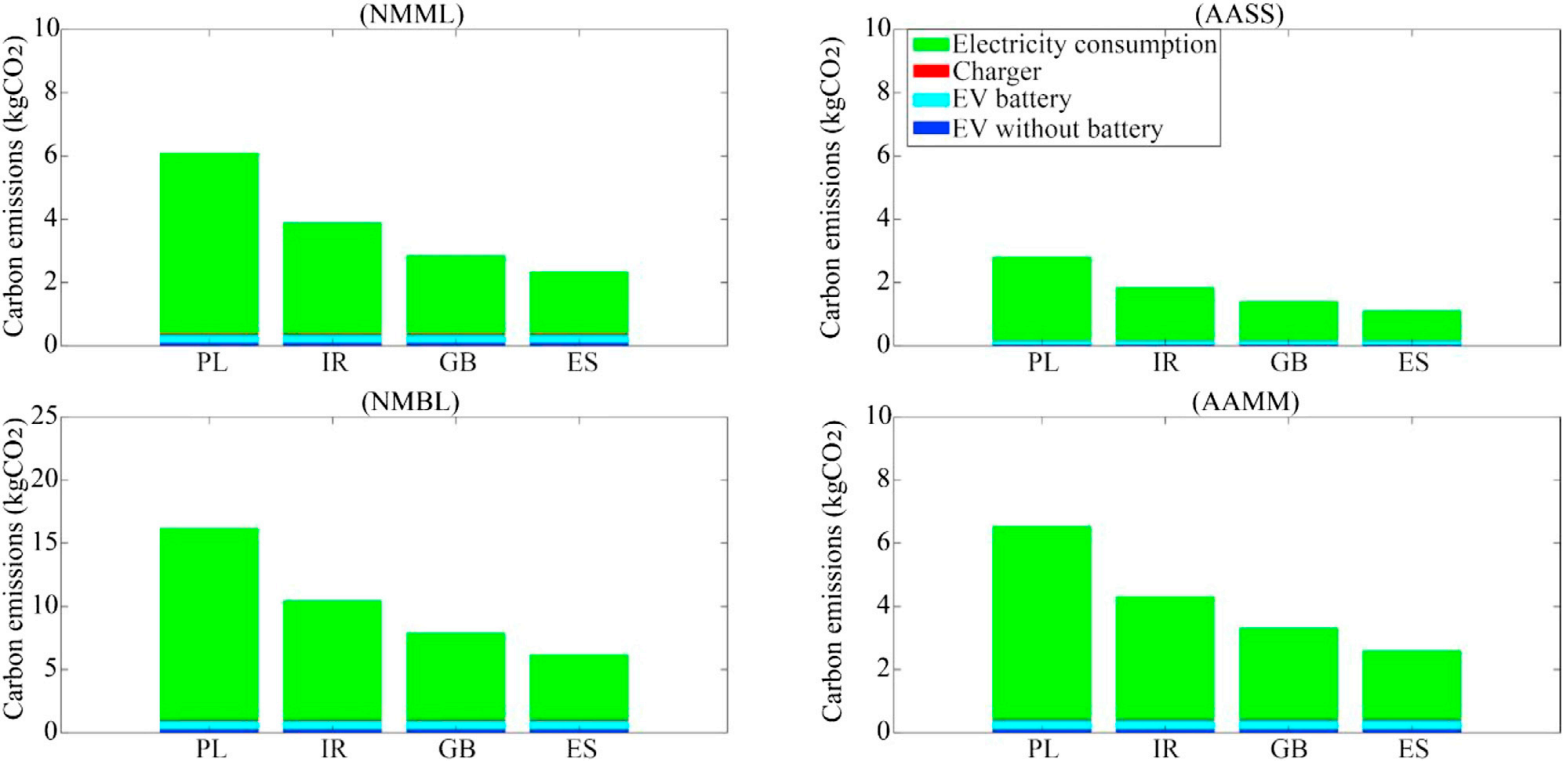
Comparison of life cycle carbon emissions of four charging behaviors in the four countries

The method used for Spain, Poland, and Ireland is the same as that used for Great Britain. By assuming that the EV charging behaviors in the three additional countries are the same as those revealed in the OLEV data for EV drivers in Great Britain, the average life cycle carbon emissions for charging behaviors in the functional unit can be compared. Figure 9 illustrates the life cycle carbon emissions of the different charging behaviors in the four countries. Poland has the highest life cycle carbon emissions, followed by Ireland, Great Britain, and Spain. The results reveal that the life cycle carbon emissions of EV charging are positively correlated with the proportion of fossil energy in the supply mix.

Table 4 shows the absolute and relative reductions in life cycle carbon emissions from smart charging for the NMML, NMBL, and AAMM behaviors in the four countries. Across all countries and charging behaviors, the greatest reductions are achieved in the two overnight charging behaviors (NMML and NMBL) in Poland, which has the highest MEFs. The MEFs in other countries are relatively low, resulting in lower emissions reductions. NMML, the charging behavior with the highest idle time, has the greatest reduction in life cycle carbon emissions from smart charging in all four countries (from 5.2% to 12.8%). The benefits of smart charging are lowest in the AAMM charging behavior across all four countries (approximately 1.5%), proving that idle time has a significant influence on the benefits of smart charging.

**Table 4:** Carbon emissions reduction from using smart charging compared with passive charging for different countries throughout the year

		Great Britain (GB)	Poland (PL)	Ireland (IE)	Spain (ES)
Charging behaviour	NMML	0.15 kg	0.78 kg	0.33 kg	0.24 kg
		5.2%	12.8%	8.4%	10.0%
	NMBL	0.40 kg	1.89 kg	0.68 kg	0.36 kg
		5.0%	11.6%	6.5%	5.8%
	AAMM	0.06 kg	0.09 kg	0.08 kg	0.04 kg
		1.9%	1.4%	1.8%	1.5%

The comparison of the four countries provides useful insights into the impact of upstream electricity emissions on the benefits of switching to EVs in different countries. The conclusions on the EV charging behaviors in Great Britain are applicable in other countries. Electricity consumption is always the main source of carbon emissions in the life cycle of EVs. The energy demand feature for charging behaviors has an impact on total carbon emissions in all countries. The benefits of smart charging are predominantly associated with idle duration.

### Interpretation and policy implications

With the rising number of charging points and financial support from the UK Government, registrations of EVs have grown rapidly in the UK in recent years and this trend is expected to continue.

Although the carbon emissions of all sessions in the datasets were not individually calculated, the life cycle carbon emissions for one charging event of the four most important charging behaviors (together accounting for more than 90% of all charging events) were studied. It was demonstrated that different behaviors have a significant impact on the emissions arising from EV charging with the current electricity supply structure in Great Britain. To maximize the benefits of increasing EV deployment, consideration should be given to policies that influence charging behavior, perhaps by linking electricity prices to current carbon intensity.

Emissions-responsive smart charging, whereby load is shifted away from periods when grid carbon intensity is high, was applied to reduce the emissions in energy consumption. Among the four charging behaviors, the overnight charging behaviors with long plug-in duration (NMML and NMBL) had the best emissions reduction performance (approximately 6% reduction in the carbon emissions from electricity consumption). AAMM with medium plug-in duration showed a 2.2% reduction proportion. NMML and NMBL, as the overnight charging behaviors, accounted for more than 50% of sessions in the dataset. Consequently, the application of EV smart charging in households could make a valuable contribution towards the decarbonization of transport.

The research was repeated for Poland, Ireland, and Spain to investigate how the results vary between different countries with different electricity systems. In general, similar results to Great Britain were achieved with smart charging during connection events with high idle times (typically those involving overnight charging) providing good emissions reduction across all four countries. A clear trend is that countries with higher emissions factors achieve greater benefits from smart charging.

This paper has presented a simulation study of the carbon emissions arising from domestic EV charging using recent data. While smart EV charging approaches offer clear emissions benefits in the current electricity systems analysed, future studies should examine the financial impacts and public acceptance of such approached, and the likely impacts of future energy scenarios. Although the idle time of public and rapid charging is estimated to be smaller than domestic charging, their environmental impact and the potential benefits from smart charging are also worthy of further investigation.

### Conclusions

Through this work we have investigated the greenhouse gas emissions from charging electric vehicles in the UK and three other European countries, and assessed the potential to reduce electric vehicle charging emissions through smart charging based on electricity grid emissions factors. The UK analysis revealed that electricity consumption accounts for the highest proportion (around 87%) of life cycle carbon emissions from EVs. These emissions vary considerably over time depending upon the share of generation from non-fossil generation, and charging behavior is the key determinant of life cycle carbon emissions. Compared with passive charging, smart charging achieves around a 6% reduction in carbon emissions when used in the most common charging behaviors, characterized by overnight charging. Plug-in duration was found to have a crucial impact on smart charging performance, with longer connections typically providing greater opportunity for load shifting and wider variations in grid emissions factors. For Great Britain, we figure out the carbon emissions of EVs under a range of charging behaviors. We also highlight the potential life cycle carbon emissions reduction brought by charging during periods when the grid mix produces relatively low emissions. The research was repeated for three other European countries; the results demonstrated that the countries with higher emissions factors achieve greater benefits from smart charging, and smart charging during connection events with high idle times (typically those involving overnight charging) provides good emissions reduction across all four countries. We expect that the findings of this study will provide a reference for the strategic implementation of electric vehicles and smart battery charging systems in the UK and the methods employed are applicable to any country.

### Limitations of the study

In this study, we note that the large-scale emission responsive charging will change the condition of the net demand, which in turn will affect the MEFs at that time. Future work could also consider the effects of charging state and examine the financial impacts and public acceptance.

## STAR★Methods

### Key resources table

**Table:** 

REAGENT or RESOURCE	SOURCE	IDENTIFIER
**Deposited data**		

OLEV domestic EV charging dataset	UK The Department for Transport	https://www.gov.uk/government/statistics/electric-chargepoint-analysis-2017-domestics.
Generation and demand data for energy supply in Great Britain	Elexon portal and Sheffield University	https://www.gridcarbon.uk
ENTSO-E dataset	ENTSO-E Transparency Platform	https://transparency.entsoe.eu/dashboard/show

**Software and algorithms**		

SimaPro 7.0	PRé Sustainability B.V.	https://simapro.com/

### Resource availability

#### Lead contact

Further information and requests for resources and materials should be directed to and will be fulfilled by the lead contact, Xueliang Yuan (yuanxl@sdu.edu.cn).

#### Materials availability

No materials were used in this study.

### Methods details

This work was focused on a large domestic charging dataset for the UK in 2017 and electricity generation data for Great Britain in 2019. The Gaussian mixture model clustering approach was used to focus the analysis on the most important charging behaviours. The emissions arising from electric vehicle charging were determined using marginal emissions factors derived from linear regression of the generation data. The GWP100 method is used to convert the LCI of the EV facilities to carbon emissions for the charging behaviours.

#### Goal and scope definition

We present an investigation into the life cycle carbon emissions of EV charging in British households in 2019. The scope of the study includes the current electricity generation mix in Great Britain and an assessment of the impact of changes to this mix combined with different charging behaviours. The functional unit of the system is taken as a single domestic EV charging event, and all analyses use the same functional unit for comparison. The system boundary is defined as “cradle to grave”. This encompasses the lifetime of the product from the extraction of raw material to waste disposal, including the impact of the charger, the battery, and the vehicle.

#### Life cycle inventory

The production, use, and disposal of the infrastructure of EVs (the charger, the battery, and the vehicle without battery) are involved in this study, with details provided in supplemental information (Table S1). The environmental impact of the infrastructure is divided by the energy consumption. The life cycle inventory of EV manufacture refers to the electric passenger car without battery in the Ecoinvent database from SimaPro 7.0 (2021). The recycling of tires, the replacements of several components and the EVs without battery is considered and the detailed recycling situation is based on the research of (Hao et al., 2017). The Li-ion (LiMn_2_O_4_) technology is chosen for the battery due to its extensive use in the EVs (Miao et al., 2019). The charger capacity is assumed to be 7 kW in this study, and the effect of varying the charger capacity from 3 kW to 22 kW is explored. Inverter and charge efficiencies are assumed as 97.0% and 98.5% respectively (Sun et al., 2019). Battery self-discharge is disregarded in this study; we believe this is a reasonable simplification, as self-discharge rates for Li-ion batteries are generally low (Zhang et al., 2020) and EVs are typically used shortly after charging.

#### Life cycle impact assessment

The GWP100 method of the ReCiPe midpoint (H) model from SimaPro 7.0 (2021) is used to convert the LCI of the EV facilities (EV without battery, battery and charger) to carbon emissions for the charging behaviours. The GWP100 value is provided by IPCC, Intergovernmental Panel on Climate Change (2020), which is defined as the greenhouse effect of various gases corresponds to the mass of CO_2_ with the same effect within a 100-year time frame.

The AEFs for the GB electricity grid in 2019 as given in the Greenhouse gas reporting (BEIS, The Department for Business, Energy & Industrial Strategy, 2019a; 2019b) are used to calculate the carbon emissions of electricity consumption for baseline analysis. The MEFs are used to calculate the carbon emissions of electricity consumption and the potential carbon emissions reduction from EV smart charging.

#### Clustering methodology

Household charging behaviour clusters are examined using a domestic EV charging dataset provided by the UK Government’s Office for Low Emission Vehicles (OLEV) (DfT, The Department for Transport, 2018). We use this dataset to model household charging behaviours focusing on the characteristics of charging time, charging duration, and energy demand. The dataset does not include any information on the chargepoint users, therefore charging frequency and driver behaviours are disregarded in the research, although they would have little impact on our results (Momtazpour et al., 2012).

The Gaussian mixture model (GMM) approach was used for clustering following the analysis of Helmus et al. (2020). GMM uses a probabilistic assignment of data points to perform clustering (Liu et al., 2018). Each cluster corresponds to the Gaussian (normal) distribution of its input elements, and the sum of the clusters conforms to the overall distribution of the original data (Patel and Kushwaha, 2020). This study assumes the presence of multiple probability distributions in the dataset, with probability distribution corresponding to a specific type of charging behaviour. In GMM, it is assumed that a dataset with observations $\;X=\left\{x_{1},x_{2},x_{3}\ldots x_{n}\right\}$ with $X\in R^{d}\left(d=4\;in\;our\;work\right)$ is generated by a mixture of K components. A component represents a clustering result of our dataset. The probability distribution of the GMM is

(Equation 1) $$p\left(x|\theta \right)=\sum_{k=1}^{K}\alpha_{k}p_{k}\left(x|\theta_{k}\right)\;$$

where X is the charging session, $p_{k}\left(x|\theta_{k}\right)$ is the Gaussian distribution density function of the kth component and $\alpha_{k}\left(\alpha_{k}\geq 0\;\&\sum_{k=1}^{K}\alpha_{k}=1\;\right)$ is the probability that the observation data belongs to the kth component (Helmus et al., 2020). For the kth component, $\theta_{k}$ is the mean and covariance matrix of the kth component, as $\theta_{k}=\left(\mu_{k},\;{\sigma_{k}}^{2}\right)$. For the four-dimensional data in our work, the Gaussian distribution of the kth components obeys the density function of

(Equation 2) $$p_{k}\left(x|\theta_{k}\right)=1/{\left(2\pi \right)}^{d/2}\sigma_{k}^{-1}exp\left\{-\frac{1}{2}{\left(x_{i}-\mu_{k}\right)}^{T}\sigma_{k}^{-2}\left(x_{i}-\mu_{k}\right)\right\}$$

The Expectation-Maximisation (EM) algorithm is widely used to estimate parameter $\theta_{k}$ (Fraley and Raftery, 2007). Because the parameters $\theta_{k}$ and $\alpha_{k}$ must be estimated and the data in each category obeys a normal distribution, Bayes' Theorem is used to modify the form of Equation (1) as

(Equation 3) $$p\left(x|\alpha ,\theta \right)=\;\sum_{k=1}^{K}\alpha_{k}p_{k}\left(x|\theta_{k}\right)$$

The new k-dimensional latent variable Z is introduced to describe which component $x_{i}$ comes from. Z should satisfy $z_{k}\left(1\leq k\leq K\right)\;$. $z_{k}$ can only take 0 or 1 and $\sum_{k=1}^{K}z_{k}=1.$ For example, if the dataset consists of 3 components and $x_{i}$ comes from the 2^nd^ component, the Z for $x_{i}$ is Z=(0,1,0). Then, $p\left(z_{k}=1|x_{i}\right)$ can represent the probability (posterior probability) that $x_{i}$ is generated by the kth component, as $p\left(z_{k}=1|x_{i}\right)=\alpha_{k}$. $p\left(z_{k}=1|x_{i}\right)$ is simply written as $\gamma \left(z_{ik}\right)$. For the Expectation step, the parameters $\theta_{k}$ and $\alpha_{k}$ are estimated by Equation (4).

(Equation 4) $$\gamma \left(z_{ik}\right)=\;\frac{\alpha_{k}p_{k}\left(x_{i}|\mu_{k},{\sigma_{k}}^{2}\right)}{\sum_{j=1}^{K}\alpha_{j}p_{k}\left(x_{i}|\mu_{j},{\sigma_{j}}^{2}\right)}=\;p\left(z_{k}=1|x_{i}\right)$$

In Equation (4), the iterative method is used to calculate $\theta_{k}$ and $\alpha_{k}$, that is, take the parameter values of $\theta_{k}$ and $\alpha_{k}$ obtained in the previous iteration (the first iteration takes their initial values). When the iteration ends, the cluster ${\text{λ}}_{i}\;$ to which $x_{i}$ belongs is determined by

(Equation 5) $${\text{λ}}_{i}=arg\;max\;\gamma \left(z_{ik}\right)\;k\in \left\{1,2,\ldots ,K\right\}$$

For the Maximisation step, the latest parameters of $\mu_{k},\;{\sigma_{k}}^{2}$ and $\alpha_{k}$ are calculated based on the value of $\gamma \left(z_{ik}\right)$ from Equation (4), as shown in Equations (6), (7) and (8), and then the next iteration can be carried out using these new estimates. The E step and M step are repeated until convergence is reached. According to the result of the EM algorithm, the charging sessions can be allocated following the cluster number.

(Equation 6) $$\mu_{k}=\frac{\sum_{i=1}^{N}\gamma \left(z_{ik}\right)x_{i}}{\sum_{i=1}^{N}\gamma \left(z_{ik}\right)}$$

(Equation 7) $${\sigma_{k}}^{2}=\frac{\sum_{i=1}^{N}\gamma \left(z_{ik}\right)\left(x_{i}-\mu_{k}\right){\left(x_{i}-\mu_{k}\right)}^{T}}{\sum_{i=1}^{N}\gamma \left(z_{ik}\right)}$$

(Equation 8) $$\alpha_{k}=\frac{1}{N}\sum_{i=1}^{N}\gamma \left(z_{ik}\right)$$

The Bayesian Information Criterion (BIC) is chosen to determine the optimal number of clusters and avoid overfitting (Equation (9)). The independent parameter M is adapted from the work of Scrucca et al. (2016). The minimum BIC value over clustering from 1 to GMM_max_ represents the best clustering (Schwarz, 1978).

(Equation 9) BIC=ln(n)M−2lnp(X,Z|θ,α)

#### Marginal emissions factors calculation

Generation and demand data provided by the GB electricity system operator, National Grid ESO, are used to analyse the carbon emissions factors of energy supply (Gridwatch, 2019). Carbon intensity factors from GridCarbon (2019) and the carbon intensity of electricity traded from overseas (Moro and Lonza, 2018) (shown in Table S2) were used to calculate national-level, one-hour resolution MEFs. The ENTSO-E Transparency Platform (2019) was used to calculate the marginal carbon emissions factors in Poland, Ireland, and Spain, as it includes hourly generation mix data for each country in Europe.

Data on electricity generation by type, total electricity demand, and interconnector flows (all in MW) for the year 2019 were obtained at five-minute resolution from Gridwatch (2019). These were averaged to hourly resolution to reduce the impact of missing points in the five-minute resolution data. The generation and demand data are further filtered by system demand net of wind and solar generation.

The adapted Hawkes's method (2010) was applied to calculate MEFs. Firstly, the system net demand (i.e., demand net of wind generation) and generator types corresponding to net demand are used for linear regression. That is, the data is binned by system net demand (shown in Figure S1, with bin widths of 2 GW). Secondly, along with binning the data by net demand, they are further binned by 60 days centred on the hour of interest. That is, rather than a linear regression analysis on the entire system data in 2019, the MEF for any given hour is calculated using the generation and net demand data for 30 days before and after. Thus, it is possible to reveal seasonal effects, particularly the reduced output from coal-fired generation in summer. The MEF at any given net demand could then be calculated using the relationship between carbon emissions and system net demand (Equations 10 and 11).

(Equation 10) $$C_{t}=\sum_{i}\left(G_{it}F_{i}\right)$$

(Equation 11) $$\Delta C_{t}=m_{t}\Delta D_{t}$$

In Equation 10, $C_{t}$ is the total carbon emissions at time *t*, $G_{it}$ is the generation from technology type *i* at time t and $F_{i}$ is the carbon intensity factor of technology type *i*. In Equation 11, $\Delta C_{t}$ is the change in emissions, $m_{t}$ is constant MEF at time t and $\Delta D_{t}$ is the change in demand.

Figures S1 and S2 show the example of the linear regression results for GB and the three countries respectively, which is the example over the period from 01/01/2019 00:00 to 02/03/2019 01:00 binned by system net demand (blue curves) to calculate the MEFs for the hour of 31/01/2019 00:00–01:00 of the linear regression approach results.

The MEF time series of GB, shown in Figure S3, has several features of note. First, comparing MEFs in 2019 with those derived by Hawkes (2010) for the period 2002–2009 reveals a significant reduction in MEFs due to low-carbon energy (particularly hydro and biomass) replacing fossil generation at the margin. Second, the MEFs are higher in winter than in summer, due to the decreased use of fossil generation in summer.

#### Charging system model

The connection and disconnection times for each cluster are rounded up to the nearest hour to match the hourly resolution of the MEF data. The charging and idle durations of each behaviour are calculated in accordance with the energy demand of each behaviour and the charger rate. This is a simplification, but the characteristics of the EV battery, charger rate, and SOC are not readily available, and thus, the influence of these components was disregarded in this study.

Two charging strategies are investigated. In the passive charging strategy, the battery starts charging from the connection time (T_conn_). In the smart charging strategy, charging occurs at the times of lowest MEF in the connection period, assuming that the charging control system has perfect foresight of MEFs in the connection period and the EV's disconnection time (T_disconn_). In reality this is not the case, therefore this analysis provides an upper limit on the potential to reduce emissions using EV smart charging. Example charging power profiles for passive and smart charging during one charging event are shown in Figure S4.

## Data Availability

This paper does not report original code, which is available for academic purposes by request from the lead contact. Links of the source data used in this paper are available in the key resources table. Data on the LCI are available within the supplemental information. Any additional information required to reanalyse the data reported in this paper is available from the lead contact upon request.

## Footnotes

Supplemental information can be found online at https://doi.org/10.1016/j.isci.2021.103499.
