# Supplementary material for: Reducing the life cycle environmental impact of electric vehicles through emissions-responsive charging
Source: iScience. 2021 Nov 22;24(12):103499. doi: 10.1016/j.isci.2021.103499 (PMC8649797; doi:10.1016/j.isci.2021.103499)

iScience, Volume *24*

Supplemental information

Reducing the life cycle environmental impact of electric vehicles through emissions-responsive charging

Yuzhou Tang, Tim T. Cockerill, Andrew J. Pimm, and Xueliang Yuan

Supplemental information

Reducing the life cycle environmental impact of electric vehicles through emissions-responsive charging

Yuzhou Tang, Tim T Cockerill, Andrew J Pimm, Xueliang Yuan

Table S1. Inventory detail of the EV facilities. (Related to STAR Methods)

Components	Life span	Data source	Data source of waste disposal
EV	2 million km [a]	Ecoinvent database	Hao et al. 2017 [b]
Li-ion battery	150,000 miles [c]	Ecoinvent database	Raugei and Winfield 2019 [d]
Charger	4000 kWh [e]	Ecoinvent database	Zhang et al. 2019 [e]

a BBC News. Tesla battery supplier Catl says new design has one million-mile lifespan. Available online: https://www.bbc.com/news/technology-52966178. [Accessed: 14 June 2020].

b Hao, H., Qiao, Q., Liu, Z., Zhao, F., 2017. Impact of recycling on energy consumption and greenhouse gas emissions from electric vehicle production: The China 2025 case. Resour Conserv Recycl. 122, 114-125.

c The tesla. Available online: https://www.tesla.com/. [Accessed: 14 June 2020].

d Raugei, M., Winfield, P., 2019. Prospective LCA of the production and EoL recycling of a novel type of Li-ion battery for electric vehicles. J Clean Prod. 213, 926-932.

e Zhang, Z., Sun, X., Ding, N., Yang, J., 2019. Life cycle environmental assessment of charging infrastructure for electric vehicles in China. J Clean Prod. 227, 932-941.

Table S2. Carbon intensity factors. (Related to STAR Methods)

Fuel Type	Carbon Intensity (gCO_2/kWh)
Biomass	120
Coal	937
Dutch Imports	474
French Imports	53
Belgian Imports	239
Gas (Combined Cycle)	394
Gas (Open Cycle)	651
Hydro	0
Irish Imports	458
Nuclear	0
Oil	935
Other	300
Pumped Storage	0
Solar	0
Wind	0

Figure S1. MEF results of linear regression for GB from 01/01/2019 00:00 to 02/03/2019 01:00. (Related to STAR Methods) (a) Example of the linear regression approach used to calculate MEFs. **(b)** Emissions factors binned by system net demand (blue curves), along with the probability of system net demand (red bars).

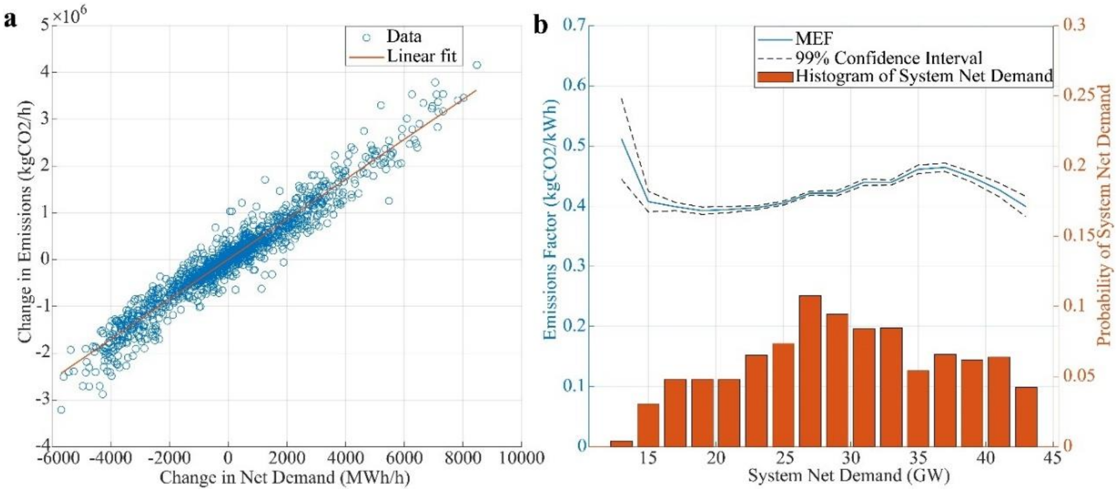

Figure S2. Results of the linear regression for the three countries. (Related to STAR Methods)

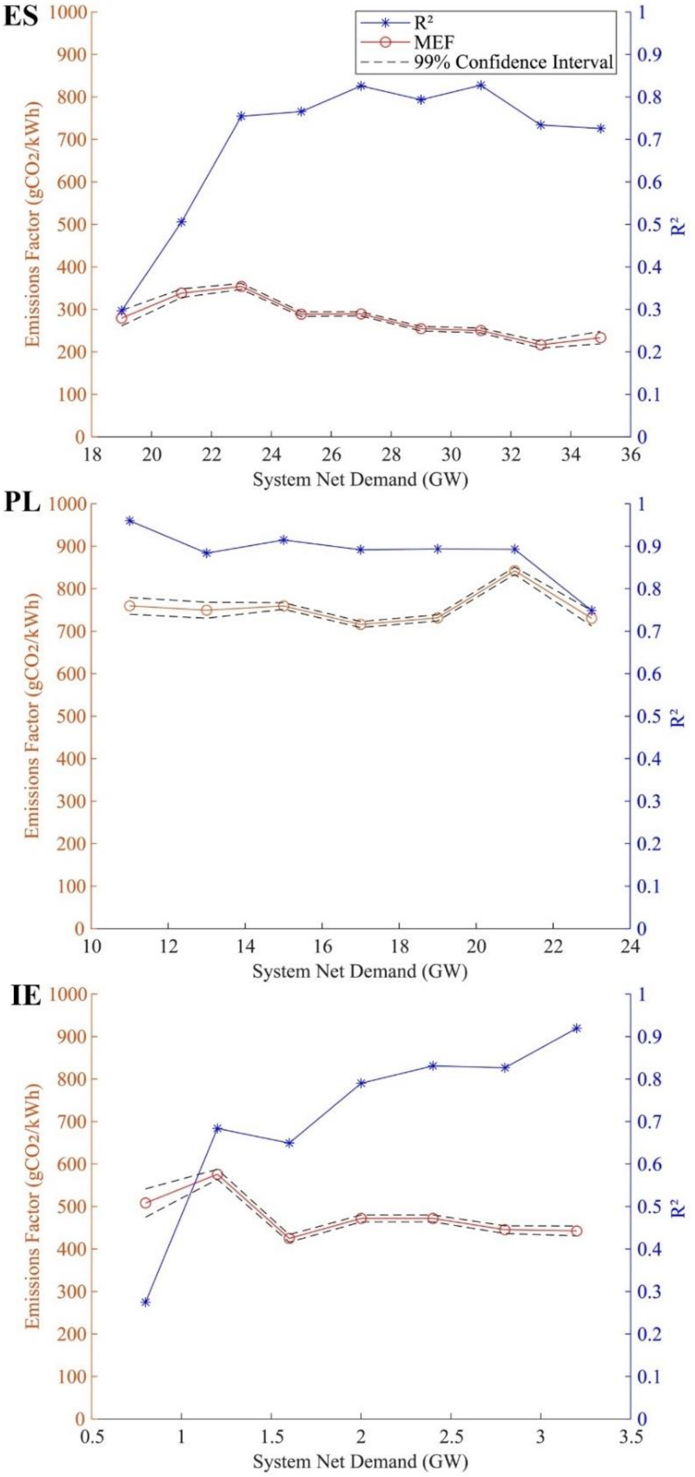

Figure S3. MEF results in 2019. (Related to STAR Methods)

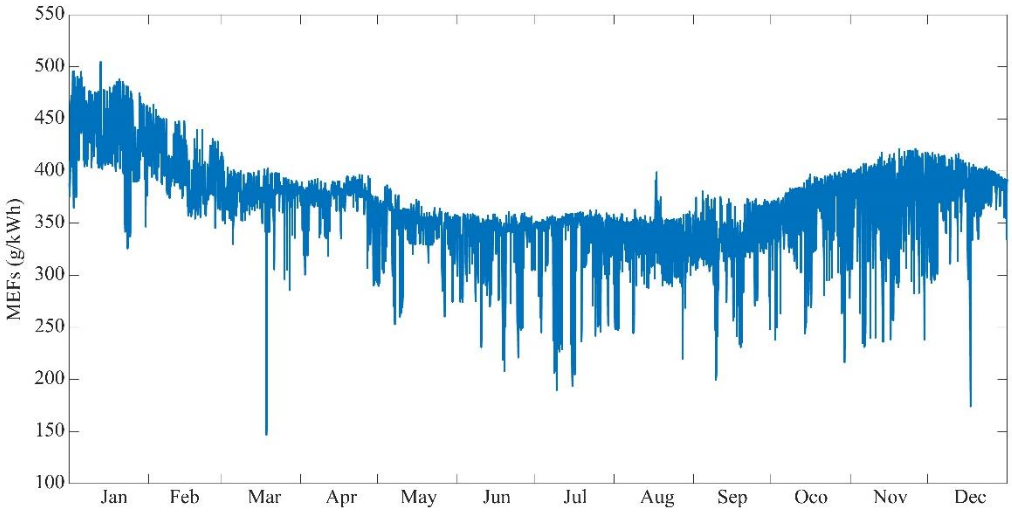

Figure S4. EV charge power profiles for a single connection event showing passive and smart charging. (Related to STAR Methods)

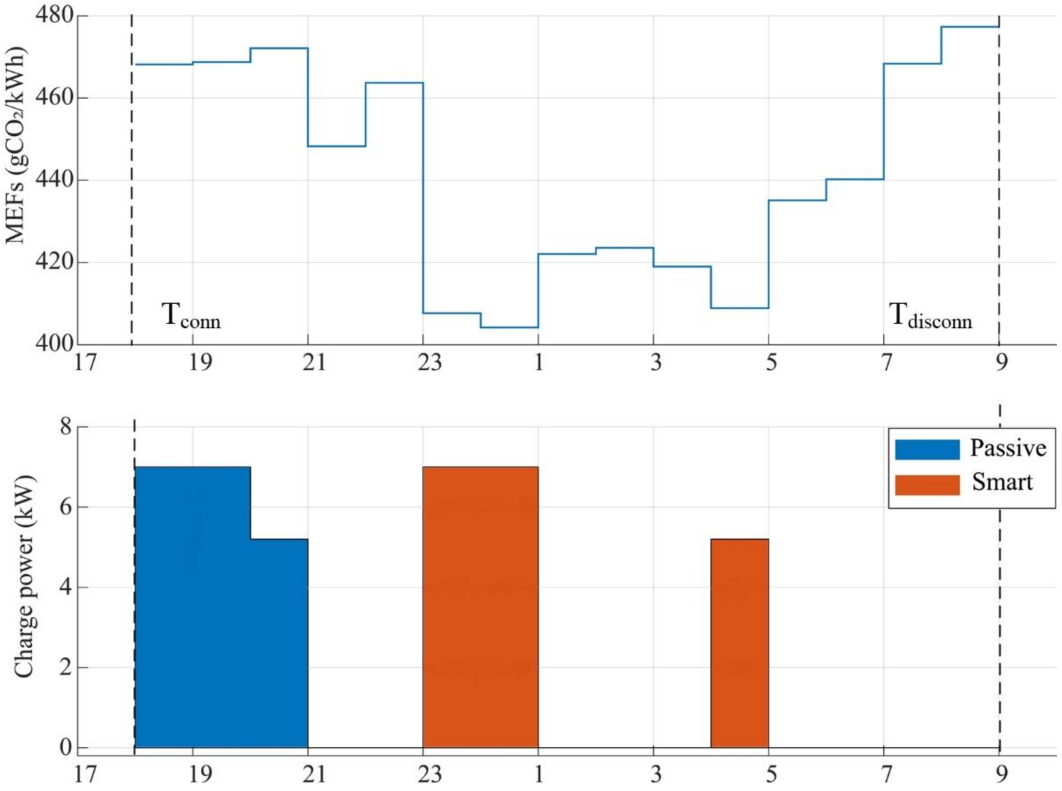

Supplement: Document S1. Figures S1–S4 and Tables S1 and S2 [file mmc1.pdf]
